# Research on Structurally Constrained KELM Fault-Diagnosis Model Based on Frequency-Domain Fuzzy Entropy

**DOI:** 10.3390/e25020206

**Published:** 2023-01-21

**Authors:** Xiaosu Feng, Guanghui Zhang, Xuyi Yuan, Yugang Fan

**Affiliations:** Faculty of Information Engineering and Automation, Kunming University of Science and Technology, Kunming 650500, China

**Keywords:** smoothing prior approach, frequency-domain fuzzy entropy, structural-constrained kernel extreme-learning machine, fault diagnosis

## Abstract

As the core equipment of the high-pressure diaphragm pump, the working conditions of the check valve are complicated, and the vibration signal generated during operation displays non-stationary and nonlinear characteristics. In order to accurately describe the non-linear dynamics of the check valve, the smoothing prior analysis (SPA) method is used to decompose the vibration signal of the check valve, obtain the tendency term and fluctuation term components, and calculate the frequency-domain fuzzy entropy (FFE) of the component signals. Using FFE to characterize the operating state of the check valve, the paper proposes a kernel extreme-learning machine (KELM) function norm regularization method, which is used to construct a structurally constrained kernel extreme-learning machine (SC-KELM) fault-diagnosis model. Experiments demonstrate that the frequency-domain fuzzy entropy can accurately characterize the operation state of check valve, and the improvement of the generalization of the SC-KELM check valve fault model improves the recognition accuracy of the check-valve fault-diagnosis model, with an accuracy rate of 96.67%.

## 1. Introduction

In the slurry pipeline transportation system, the high-pressure diaphragm pump is the core power source of the system. As an important mechanical part of the high-pressure diaphragm pump, the working condition of the check valve affects the performance of the entire system; if internal damage occurs, it will affect the operation of the whole system. Therefore, it is of great significance to monitor the operating status of the check valve [1,2].

The high-pressure diaphragm pump is a typical nonlinear dynamical system. The vibration signal generated by the check valve during operation has non-stationary and nonlinear characteristics. In recent years, the entropy theory of nonlinear dynamics methods has been widely used in the field of fault diagnosis because of its good ability to characterize fault information [3,4,5]. Cui Ruihua [6] proposed a method for extracting fault features based on variational mode decomposition-approximate entropy, combined with the support-vector machine to accurately identify arc faults. Chen K. [7] used the complementary ensemble empirical mode decomposition (CEEMD) algorithm to analyze the vehicle condition signal and used the sample entropy to eliminate the noise, which improved the reliability of the operational path analysis (OPAX) method. Chen Qiangqiang [8] used smoothness prior analysis (SPA) to adaptively decompose the rolling bearing signal into tendency items and fluctuation items and established a rolling-bearing performance-degradation-prediction model based on permutation entropy, which effectively monitored the bearing operation.

The above literature has been successfully applied to the problem of mechanical-fault diagnosis, but its popularization and application is limited by the deficiency in feature extraction of the signal, such as the deviation of approximate entropy when matching itself. Sample entropy is heavily dependent on data length and poor anti-interference, and permutation entropy is not sensitive to abnormal points in the internal sequence when calculating the probability distribution [9]. In addition, all of the above methods ignore the frequency-domain entropy characteristics of the signal. In order to overcome the above problems, the frequency-domain fuzzy entropy of SPA components is used for check-valve fault-information extraction. The SPA adaptive decomposition of the check-valve vibration signal is performed to extract the tendency and fluctuation terms of the signal, and the frequency-domain fuzzy entropy of the tendency and fluctuation terms is calculated as the characterization vector of the operating state of the check valve to achieve better feature extraction.

A reliable and stable fault-state-identification model based on the frequency-domain fuzzy entropy feature-information-extraction method of the SPA component is the key to the fault diagnosis of the check valve. The extreme learning machine (ELM), as a single hidden layer forward neural network, is widely used in the construction of fault-diagnosis models with its good nonlinear fitting ability and efficient learning efficiency [10,11,12,13]. However, the random selection of hidden nodes in ELM results in the unstable output results of the hidden layer, which reduces its generalization ability and robustness. For this reason, Zhang [14] used the L1 norm as a constraint item of the loss function and proposed an outlier robust extreme-learning machine (ORELM), which effectively improved the generalization performance of the model. Although the ORELM based on the L1 norm requires greater computational time cost, the successful application of ORELM provides a new way to improve the generalization performance of ELM. Drawing on ORELM, the paper proposes the kernel extreme-learning machine (KELM) functional norm-regularization method to construct the Structure Constrained kernel extreme-learning machine (SC-KELM) model and applies it to check-valve fault diagnosis. The introduction of the functional norm-regularization method improves the generalization ability and recognition accuracy of the model.

## 2. Basic Theory

### 2.1. Smoothing Prior Approach (SPA)

SPA is an estimation method for the smoothing tendency of nonlinear signals, which is now widely used in the geological [15] and medical [16] fields for signal processing, and its principles are mainly as follows.

If the original signal is ***Z*** and its nonlinear trend term is represented by ***Z****_t_*, construct an observation model for ***Z****_t_*.

(1) $$Z_{t}=H\theta +v$$

where $H\in R^{N\times M}$ is the observation matrix, N is the data length, $\theta \in R^{M}$ is the regression parameter, and *v* is the observation error.

In the process of solving the optimal solution $\hat{\theta }$, based on the least-squares method, a regular term factor is introduced to restrict the estimated tendency term, that is,

(2) $$E=\underset{\theta }{\arg\min}\{{\left\|H\theta -Z\right\|}^{2}+\lambda \left\|D_{d}{\left(H\theta \right)}^{2}\right\|\}$$

where *λ* is the regularization parameter and ***D****_d_* is the matrix of the *d*th order differential operator for the discretization of the original signal, and the calculation of ***D****_d_* is illustrated below in terms of the second order discrete differential operator.

If there are *N* local peaks inside the original signal, the tendency term can be expressed as **Z***_t_* = [***Z***_1_, ***Z***_2_, …, ***Z****_N_*]^T^; then,

(3) $$\begin{array}{c} Z_{t2}={\left[\begin{array}{ccc} Z_{3}-Z_{2}-(Z_{2}-Z_{1}), & \cdots & Z_{N}-Z_{N-1} \end{array}-(Z_{N-1}-Z_{N-2})\right]}^{T}\\ =\left[\begin{array}{cccccc} 1 & -2 & 1 & 0 & \cdots & 0\\ 0 & 1 & -2 & 1 & \cdots & 0\\ \vdots & \vdots & \vdots & \vdots & \vdots & \vdots \\ 0 & \cdots & 0 & 1 & -2 & 1 \end{array}\right]*\left[\begin{array}{c} Z_{1}\\ Z_{2}\\ \vdots \\ Z_{N} \end{array}\right] \end{array}$$

where the constant matrix is the representation of ***D****_d_*; by analogy with the above procedure, the solution of the discrete differential operator matrices of other orders can be achieved.

Find the derivative of Equation (2), it follows that

(4) $$\frac{\partial E}{\partial \theta }=2\theta^{T}H^{T}H-Z^{T}H+\lambda \theta^{T}H^{T}D_{d}H\theta$$

(when the above formula is equal to 0, the *θ* obtained is the optimal solution of Equation (2)).

(5) $${\hat{\theta }}_{\lambda }={\left(H^{T}H+\lambda H^{T}D_{d}^{T}D_{d}H\right)}^{-1}H^{T}Z$$

(6) $${\hat{Z}}_{t}=H{\hat{\theta }}_{\lambda }$$

where ***H*** is usually chosen to be the identity matrix $I\in R^{N\times N}$, for the matrix $D_{d}$, which gives a good estimation of the tendency term in the signal when the order is 2. It can be expressed as

(7) $$D_{2}=\left(\begin{array}{cccccc} 1 & -2 & 1 & 0 & \cdots & 0\\ 0 & 1 & -2 & 1 & \ldots & \vdots \\ \vdots & \vdots & \vdots & \vdots & \vdots & 0\\ 0 & \ldots & 0 & 1 & -2 & 1 \end{array}\right)$$

According to the above process, the fluctuation term of the original signal ***Z***_det_ is

(8) $$Z_{\det}=Z-H{\hat{\theta }}_{\lambda }=\left[I-(I+\lambda D_{2}^{T}D_{2})-1\right]Z$$

### 2.2. Frequency-Domain Entropy (FDE)

The frequency-domain entropy reflects the modal information of the signal from the perspective of the frequency-component structure and the characteristics of the signal and uses the spectrum-energy-difference coefficient to measure the complexity of the signal. The more discrete the distribution of different frequency components, the larger the entropy value, and the calculation process is as follows [17].

Given the vibration signal ***X*** = {*x*_1_, *x*_2_ …, *x_N_*}, the spectral energy sequence is obtained by processing with the Fourier transform method.

(9) $$X[K]=\left|\sum_{i=0}^{N-1}x[i]\exp(-j\frac{2\pi }{N}kn)\right|k=0,1\ldots N-1$$

Based on the definition of Shannon entropy, its frequency-domain entropy is expressed as follows.

(10) $$E_{nf}=-\sum_{k=0}^{N-1}P_{k}\ln P_{k}$$

where $P_{k}=S_{k}/\sum_{k=0}^{N-1}S_{k}$, denotes the proportion of the *k*th spectrum among all spectra.

### 2.3. Frequency-Domain Fuzzy Entropy (FFE)

Based on the calculation process of the fuzzy entropy definition of the frequency-domain entropy, the frequency-domain fuzzy-entropy algorithm is proposed. First, the spectrum component extracted from formula (9) is restructured in phase space with length m, and a total of *N*-*M*+1 vector is obtained, that is,

(11) $$\vec{X_{i}^{m}}=\{\vec{x}(i),\cdots \vec{x}(i+m-1)\}-{\vec{x}}_{0}(i)$$

where *I* = 1, 2, … *N-m*+1, ${\vec{X}}_{i}^{m}$ is a vector consisting of *m* consecutive values starting from the *i*th point minus the value of the mean ${\vec{x}}_{0}(i)$.

(12) $${\vec{x}}_{0}(i)=\frac{1}{m}\sum_{j=0}^{m-1}\vec{x}(i+j)$$

Define the distance $d_{ij}^{m}$ between ${\vec{X}}_{i}^{m}$,${\vec{X}}_{j}^{m}$ as the one with the largest difference between the corresponding elements of both.

(13) $$d_{ij}^{m}=\underset{k\in (0,m-1)}{\max}\{\left|\vec{x}(i+k)-\vec{x_{0}}(i)-(\vec{x}(j+k)-\vec{x_{0}}(j))\right|\}$$

where *i*,*j* = 1, 2, *N-m*, *i* ≠ *j*.

Calculate the fuzzy affiliation of ${\vec{X}}_{i}^{m}$, ${\vec{X}}_{j}^{m}$ based on the distance d $D_{ij}^{m}$.

(14) $$D_{ij}^{m}=\mu (d_{ij}^{m},n,r)=\exp(\frac{-(d_{ij}^{m})n}{r})$$

where *n*, *r* are the boundary gradient and width of the fuzzy function $\mu (d_{ij}^{m},n,r)$, respectively. 

Find the mean of all vague affiliations other than itself.

(15) $$\phi^{m}(n,r)=\frac{1}{N-m}\sum_{j=1}^{N-m}\frac{1}{N-m-1}\sum_{j=1,j\neq i}^{N=m}D_{ij}^{m}$$

Growing *m* to *m*+1 and repeating the above process; then,

(16) $$\phi^{m+1}(n,r)=\frac{1}{N-m}\sum_{j=1}^{N-m}\frac{1}{N-m-1}\sum_{j=1,j\neq i}^{N=m}D_{ij}^{m+1}$$

When the length of the sequence is a finite value, the frequency-domain entropy of the sequence can be expressed as follows:

(17) $$\mathrm{FFE}(n,r)=\ln\phi^{m}(n,r)-\ln\phi^{m+1}(n,r)$$

## 3. Structurally Constrained KELM (SC-KELM) 

ELM is a single hidden layer forward neural network, and the principle is as follows:

Given *N* training samples $\left(X_{i},Y_{i}\right)$, where $X_{i}=[x_{i1},x_{i2},\ldots ,x_{in}{]}^{T}\in R^{n}$, $Y_{i}=[Y_{i1},Y_{i2}\ldots ,Y_{im}{]}^{T}\in R^{m}$. If the number of hidden layer nodes of ELM is *L*, the following condition should be met in order to minimize the output error of the learning target:

(18) $$\sum_{j=1}^{L}\left\|O_{j}-Y_{j}\right\|=0$$

There is ***β****_i_*, ***W****_i,_* and $b_{i}$, such that

(19) $$\sum_{i=1}^{L}\beta_{i}g(W_{i}\cdot X_{j}+b_{i})=Y_{j},j=1,\ldots N$$

where *g* is the activation function, ***W****_i_* = [*w_i_*_1_, *w_i_*_2,…_*w_i__m_*]^T^ is the input weight and ***β****_i_* is the output weight; ***b****_i_* is the bias of the *i*th hidden layer cell; and ***W****_i_**X**_j_* denotes the inner product of ***W****_i_* and ***X****_j_*, which can be represented by the matrix as follows:

(20) Hβ=Y

where ***H*** is the output matrix of the hidden layer, ***β*** is the output weight matrix, and ***Y*** is desired output result of the learning model.

To solve the instability of output results caused by the random initialization of ELM parameters, Huang [18] proposed the kernel extreme-learning machine (KELM, kernel extreme-learning machine), which introduces a nonlinear mapping *φ* for constructing the kernel matrix ***H***_ELM_ [19,20]. The elements of ***H***_ELM_ at row *i* and column *j* are defined as follows:

(21) $$H_{\mathrm{ELM}}{}_{\left(i,j\right)}=K(x_{i},x_{j})$$

***K***(*x_i_*,*x_j_*) is generally obtained using the radial basis function:

(22) $$K(x_{i},x_{j})=\exp\left(-\frac{{\left\|x_{i}-x_{j}\right\|}^{2}}{2\sigma^{2}}\right)$$

where *σ* is the kernel function width; then, its output is

(23) $$f(x)=h(x)H^{T}{\left(\frac{I}{C}+HH^{T}\right)}^{-1}Y=\left[\begin{array}{c} K(x,x_{1})\\ \vdots \\ K(x,x_{N}) \end{array}\right]{\left(\frac{I}{C}+HH^{T}\right)}^{-1}Y$$

In order to improve the generalization performance of KELM, the paper proposes the function parametric constraint KELM algorithm to construct the SC-KELM diagnostic model. The optimization target function is as follows:

(24) $$L=\underset{\beta }{\arg\min}{\left\|H\beta -Y\right\|}^{2}+\alpha {\left\|f\right\|}_{k}{}^{2}$$

where ${\left\|f\right\|}_{k}$ is the function norm constraint term, which is the norm of the function *f* in the regenerating kernel Hilbert space (RKHS, reproducing kernel Hilbert space), and α is the corresponding penalty factor.

In the RKHS, the solution of *f* is as follows:

(25) $$f=\sum_{i=1}^{L}\beta_{i}\varphi (x_{i})$$

where *φ* is the nonlinear mapping function, the function *f* in RKHS consists of φ(xi), and *L* is the number of hidden layers.

Let ***β*** = [***β***_1_, ***β***_2_,…***β****_L_*]^T^, ***φ*** = [***φ***(***x***_1_), ***φ***(***x***_2_),…***φ***(***x****_L_*)]; then, the norm of the function f can be solved as follows:

(26) $${\left\|f\right\|}_{k}^{2}={\left\|\phi \beta \right\|}_{k}^{2}=\beta^{T}\varphi^{T}\varphi \beta =\beta^{T}K\beta$$

where ***K*** is the Gram matrix in the Hilbert space of the regenerating kernel. Substituting Equation (26) into Equation (24), we obtain

(27) $$L=\underset{\beta }{\arg\min}\beta^{T}H^{T}H\beta +Y^{T}Y-Y^{T}H\beta -\beta^{T}H^{T}Y+\alpha \beta^{T}K\beta$$

The derivation of the above equation.

(28) $$\frac{\partial L}{\partial \beta }=\alpha \beta^{T}K+\beta^{T}H^{T}H-Y^{T}H$$

When the derivative is equal to 0, the output weight matrix is obtained as

(29) $$\beta ={\left(H^{T}H+\alpha K\right)}^{-1}H^{T}Y$$

Input the test sample *x*, and the output result of the model is

(30) $$f(x)=h(x){\left(H^{T}H+\alpha K\right)}^{-1}H^{T}Y$$

## 4. Structurally Constrained KELM Fault-Diagnosis Model Based on Frequency-Domain Fuzzy Entropy

The basic process of structurally constrained KELM based on the frequency-domain fuzzy entropy is as follows:

(1) The check-valve vibration signal acquisition: to obtain the vibration signal data of the check valve under normal, stuck valve, and wear states.

(2) The nonlinear tendency and fluctuation term extraction of the vibration signal: using the SPA method to separate the nonlinear tendency and fluctuation term of the signal.

(3) The vibration signal feature vector extraction: the frequency-domain fuzzy entropy of the component signal is obtained to construct the input vector of the fault identification model.

(4) Sample partitioning: the feature vector set obtained is randomly partitioned into 50% for training and 50% for testing sets.

(5) Construct and optimize the model: calculate the ***H***_ELM_ matrix according to formula (22), set the range of α, input the training set to the SC-KELM recognition model, and calculate the output weight ***β*** according to formula (29).

(6) Input the test samples to identify the operation status of check value and evaluate the model performance in terms of accuracy of recognition.

## 5. Experiments and Analysis

### 5.1. Experimental Equipment and Data

In this paper, a high-pressure diaphragm pump station check valve of an iron ore concentrate pipeline No. 3 in Yunnan is taken as the research object, and the vibration signal data of its normal, wear, and stuck valve operation states are selected to verify the effectiveness of the scheme proposed in this paper. The names and models of the equipment are shown in Table 1.

**Figure 1:**
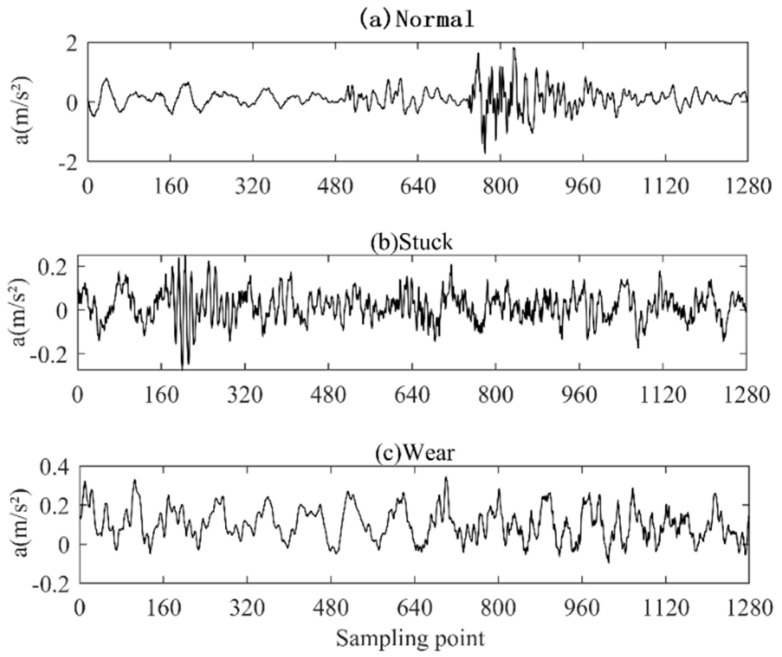
Time domain diagram of vibration signal of check valve in different working conditions.

In the process of data acquisition, the sampling frequency f of the check valve acceleration sensor is set to 2560 Hz, the data length of a single sampling is 1280, and 60 groups of single data are collected under different operating states. The vibration acceleration signals are shown in Figure 1, where a is the acceleration; the horizontal axis corresponds to the number of sampling points, from which it can be seen that in the normal operating state, its signal is relatively smooth. Occasionally, there will be certain fluctuations, mainly by the noise from the outside, but when its operating state is the stuck or wear state, the signal contains shock characteristics.

### 5.2. Feature Extraction

**Figure 2:**
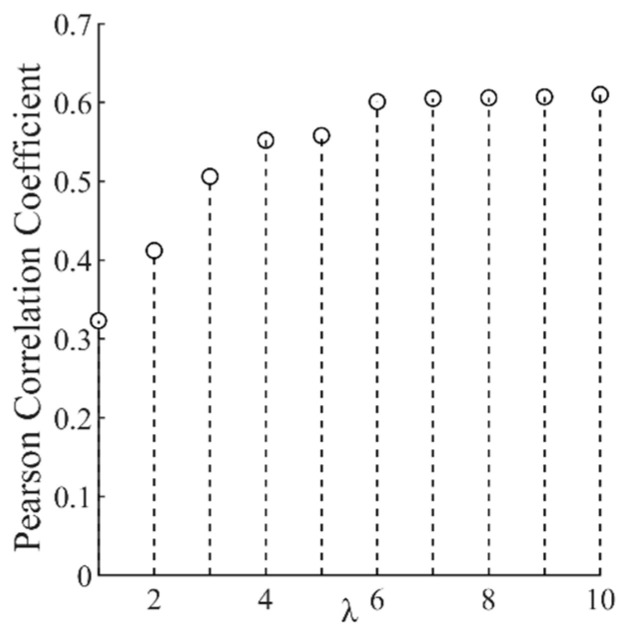
Pearson coefficient of tendency item and original signal.

**Figure 3:**
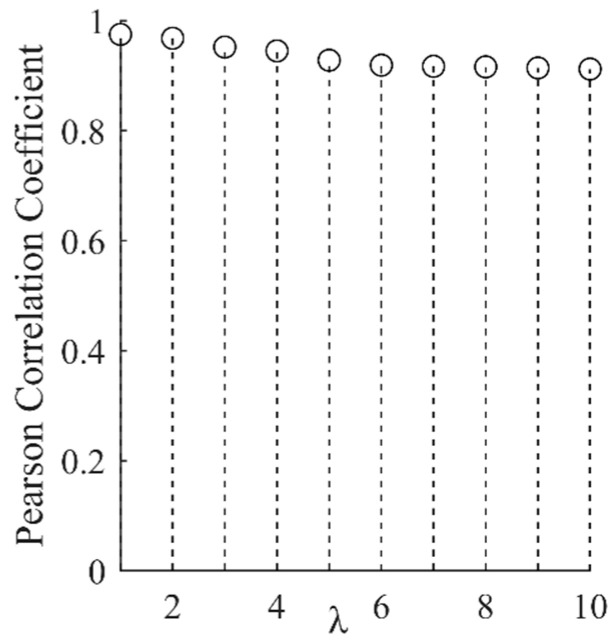
Pearson coefficient of fluctuation item and original signal.

When extracting the nonlinear tendency and fluctuation terms of vibration signals with SPA, λ will bring some influence on the results. Hence, a randomly selected section of the stuck signal with different coefficients λ was subjected to SPA decomposition, and the correlation coefficient was used to measure the level of separation of the tendency and fluctuation term from the original signal, as shown in Figure 2 and Figure 3.

**Figure 4:**
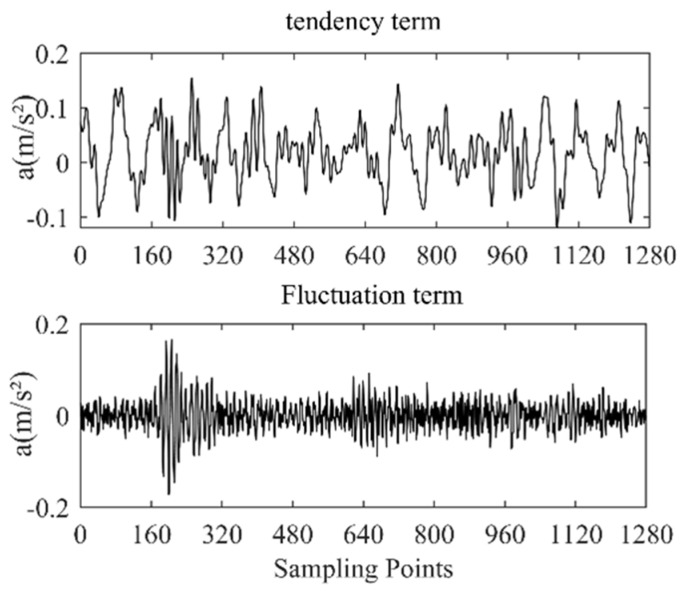
SPA decomposition results of the stuck valve signal.

From Figure 2 and Figure 3, it can be seen that when λ = 6, the separation of the tendency term, the fluctuation term, and the original signal tends to be stable; if the value of λ is too large, the extraction of the tendency term will be too aggressive, while if it is too small it will be too smooth and reduce the distinction between different states [16], so we set λ = 6. The result of the SPA decomposition is shown in Figure 4.

It can be seen from Figure 4 that after SPA decomposition, the obtained tendency item and fluctuation item are clearly distinguished. They reflect the basic characteristics of the vibration signals from different aspects. The tendency items accurately synchronize the curve trajectory of the original signal, the fluctuation items record the original detailed information such as signal amplitude and peak value, and the decomposition results illustrate the rationality of the SPA’s signal decomposition.

**Figure 5:**
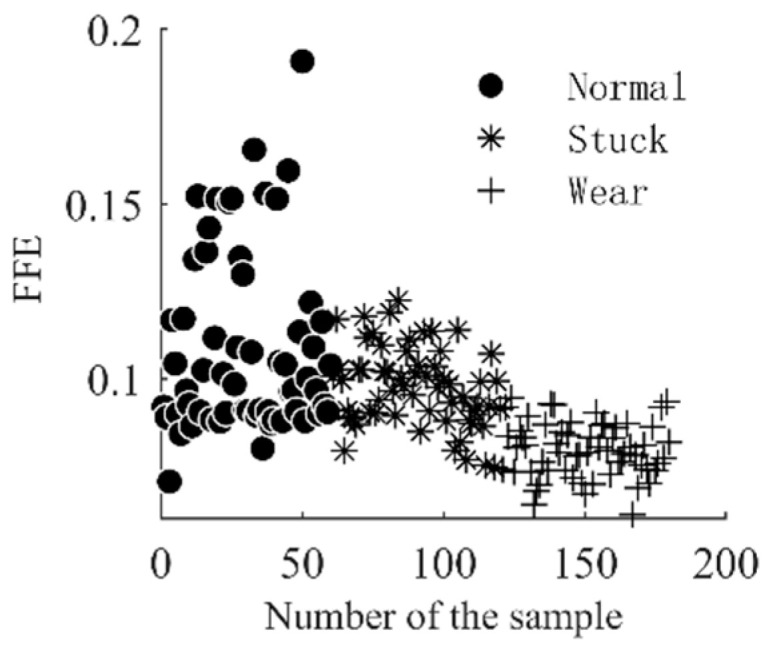
FFE of tendency item of check valve under different conditions.

**Figure 6:**
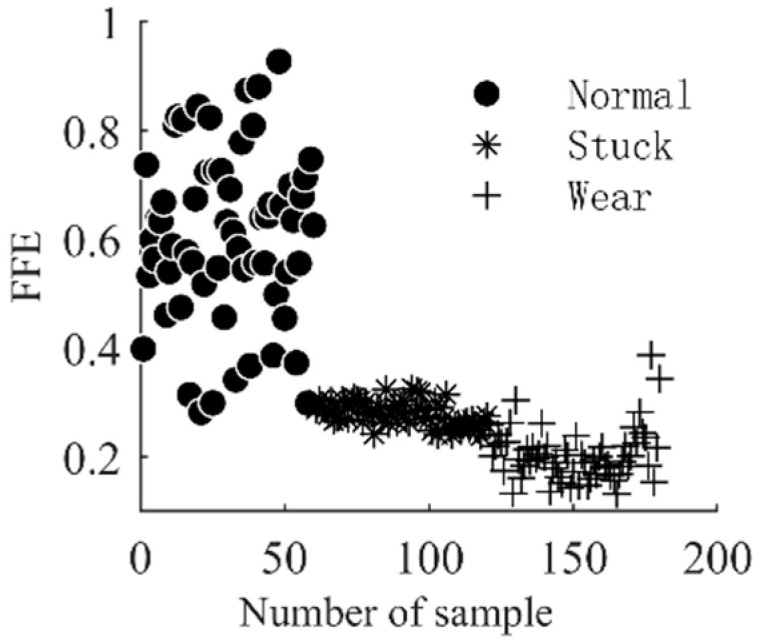
FFE of fluctuation item of check valve under different conditions.

After the separation of the nonlinear trend and fluctuation terms of the check-valve vibration signal, the FFE value of the corresponding component is used the input vector of the diagnostic model. The solution process of FFE mainly includes phase-space reconstruction and fuzzy membership calculation, where the dimension m in phase-space reconstruction, the boundary width r of the fuzzy affiliation function in the affiliation calculation process, and the gradient n will bring an impact on the results. The value of m is generally 2/3 in order to effectively ensure the integrity of the sequence information; if r is too large it will cause the loss of information in the signal, and if it is too small it will reduce the noise resistance. Usually, it is set to 0.15–0.25SD (SD is the standard deviation of the sequence), while n mainly plays a weighting role in the similarity calculation process of the reconstruction vector, generally consistent with the dimension of the reconstruction component [21], so this paper sets m = 2, r = 0.2 SD, n = 2 in turn. According to the above settings, a total of 3 groups * 60 frequency-domain fuzzy entropy vectors are obtained, and their distribution is shown in Figure 5 and Figure 6. As can be seen from the figure, from the perspective of the components, the difference in entropy values between the fluctuation term and the trend term is obvious; this is because the entropy measures the degree of complexity and regularity of the signal; the tendency term depicts the basic physical characteristics of the vibration signal, which is relatively smooth and regular, so the value of entropy is smaller; the fluctuation term specifies the change process of the vibration signal in the adjacent time period; and the sharpness of its change is much larger than that of the tendency term, so there are obvious differences in entropy values between the two components. From the entropy value of the different operating conditions of the check valve, whether it is the tendency term or the fluctuation term, the entropy value in different operating states has good stratification in spatial distribution. Therefore, the frequency-domain fuzzy entropy can be used to complete the fault information extraction of the vibration signal of the check valve, and the effective combination of the frequency-domain fuzzy entropy of the trend term and the fluctuation term can be used to characterize the different operating states of the check valve.

### 5.3. Operational-Status Recognition

**Figure 7:**
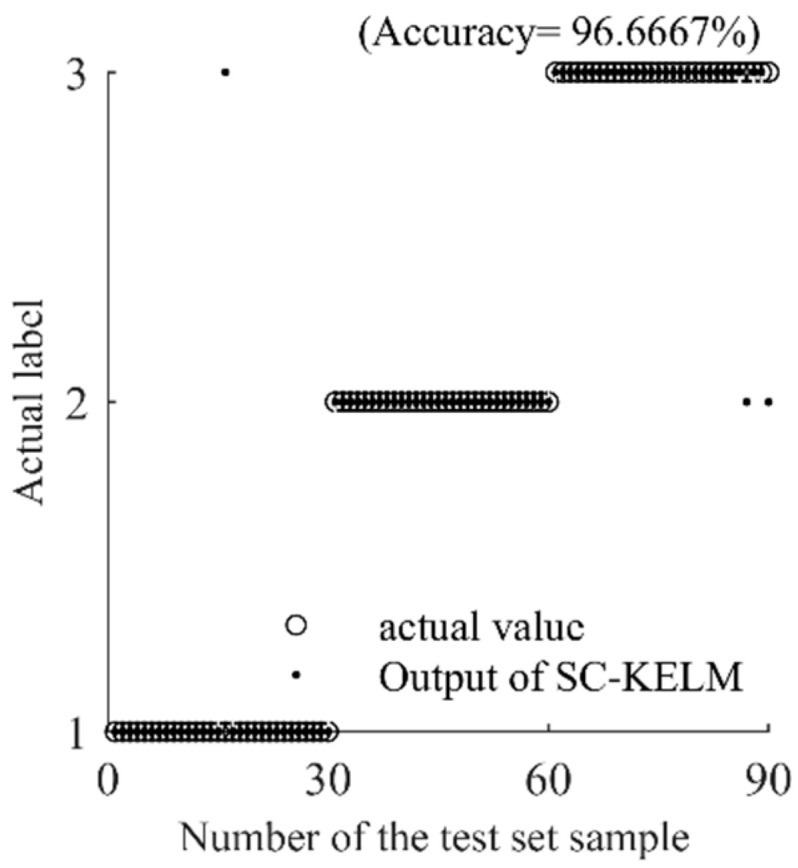
Fault diagnosis results of the model.

After the above processing, the effective combination of the frequency-domain fuzzy entropy of the extracted nonlinear trend item and fluctuation item is used as the input vector of the fault-diagnosis model. First, 30 samples are randomly selected in the fault-feature sample set of each state as training samples, and the remaining 30 samples are used as test samples. The labels of normal, stuck valve, and wear state correspond to “1”, “2”, and “3” respectively; secondly, the training samples are sent to the SC-KELM model for training, and the output weight matrix is obtained; finally, the test samples are used to verify the model, and the diagnostic results are shown in Figure 7. It can be seen from the figure that the fault-identification accuracy rate of the model is 96.67%, which proves the effectiveness of the scheme proposed in this paper.

In order to illustrate the extraction performance of the fault features, SPA-FFE, EMD-FE, EMD-FFE, and SPA-FE are used for fault-feature extraction and compared with the proposed scheme in this paper, respectively. After EMD decomposition, 10 intrinsic mode functions are obtained from the original signal of each operating state. It is generally believed that the IMF, which is highly correlated with the original signal, contains adequate information of the fault feature. Therefore, the correlation coefficients between multiple IMFs and the original signal are solved separately, and the results are shown in Table 2. It can be seen from the table that IMF1, IMF2, and IMF4 in the normal state maintain a high correlation with the original signal, while the wear state is IMF3, IMF8, and IMF9, respectively, and in the stuck valve state, the first three-order IMF and the original signal has a high Pearson coefficient, so the FFE and FE of the IMF obtained from the above decomposition are used to characterize the operating state of the check valve. Finally, the eigenvectors extracted under the different schemes were input into SC-KELM for fault identification, and the results are shown in Table 3. It can be seen from the figure that both the local fault-tolerance rate under the same working conditions and the overall recognition accuracy have been improved to a certain extent by the scheme in this paper.

**Figure 8:**
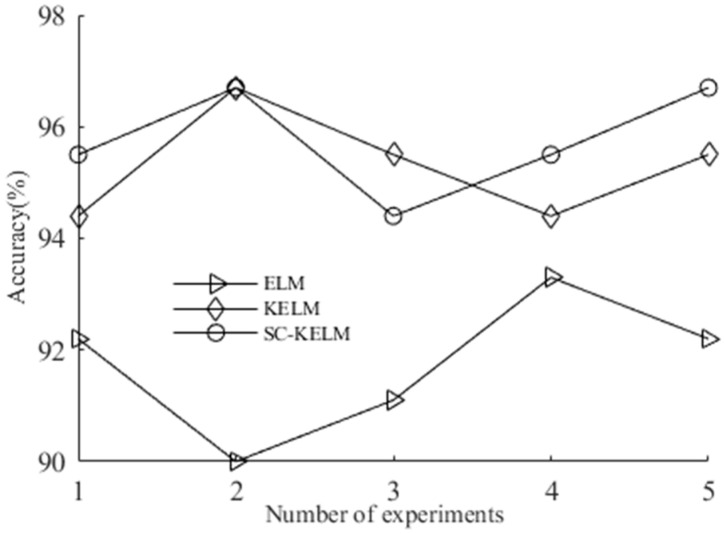
Fault-state recognition accuracy of different diagnostic models.

Meanwhile, to illustrate the generalization performance of SC-KELM, the fault features of the check valve extracted by SPA-FFE are used as the input vectors of the ELM and KELM, respectively, and the diagnostic results are compared with MC-KELM. The ELM selects the Sigmoid activation function; the number of hidden layer nodes is set to 30, and the regulation parameters in MC-KELM and KELM are set to 0.5. During the experiment, the training and testing samples were randomly selected, the ratio remained unchanged, and each algorithm was run independently 5 times; its recognition accuracy curve is shown in Figure 8, from which it can be seen that the recognition accuracy of SC-KELM is significantly higher that the ELM and KELM, and the stability of the SC-KELM recognition rate is better than the KELM because the regular term of the function can more effectively control the range of the output weight and enhance the stability and generalization ability of the model.

## 6. Conclusions

A function norm regularization method suitable for the KELM is proposed, which is used to constrain the structure of KELM, and the SC-KELM check-valve fault-diagnosis model is established based on the regularized KELM. The nonlinear dynamics of the check valve are characterized by the SPA component frequency-domain fuzzy entropy to characterize the operating state of the check valve, which is used to train the SC-KELM fault-diagnosis model, and the model is applied to the fault-diagnosis problem of the check valve of the high-pressure diaphragm pump; the results show that:

(1) The SPA algorithm adaptively decomposes the vibration signal to obtain the tendency item and fluctuation item of the vibration signal, which can effectively separate the nonlinear tendency in the signal and highlight the signal component characteristics; it is beneficial for extracting the frequency domain characteristics of the vibration signal of the check valve.

(2) The frequency-domain fuzzy-entropy algorithm proposed can measure the frequency-domain complexity of the vibration signal and effectively extract the fault information contained in the vibration signal of the check valve. The SPA component frequency-domain fuzzy entropy can accurately characterize the operating state of the check valve.

(3) The SC-KELM fault-diagnosis model is established based on the frequency-domain fuzzy entropy because its structure is constrained by the functional norm, and the generalization performance has been improved. Applying the model to the fault problem of the check valve, the accuracy of the diagnosis reaches 96.67%, which verifies the effectiveness of the model.

## Figures and Tables

**Table 1:** Equipment name and model.

Device Name	Model Number
Three cylinder reciprocating pump	TZPM
Acceleration transducer	PCB-ICP
Data acquisition cards	PXIe-3342
controller	PXI-3050EXT 2.7 GHZ
Industrial personal computer	PXI-9108EXT 8-Slot PXI Chassis

**Table 2:** Correlation coefficient between IMF and original signal.

IMF	Normal	Wear	Stuck
IMF_1_	0.4792	0.2644	0.4214
IMF_2_	0.2958	0.3846	0.4093
IMF_3_	0.2289	0.6064	0.6345
IMF_4_	0.3125	0.2623	0.2963
IMF_5_	0.0450	0.0169	0.0095
IMF_6_	0.0190	0.0089	0.0123
IMF_7_	0.0004	0.0535	0.0090
IMF_8_	0.0001	0.3865	0.1026
IMF_9_	0.0089	0.4123	0.1894
IMF_10_	0.0019	0.1207	0.1002

**Table 3:** Diagnostic accuracy corresponding to different feature extraction schemes.

Feature Extraction Scheme	Total Number of Samples/Number of Misdiagnoses			Overall Accuracy
	Normal	Stuck	Wear	
SPA-FFE	30/1	30/0	30/2	96.67%
SPA-FE	30/2	30/1	30/2	94.44%
EMD-FFE	30/2	30/2	30/2	93.33%
EMD-FE	30/2	30/3	30/3	91.11%

## Data Availability

The data used to support the findings of this study are available from the corresponding author upon request.
